# Work-Related Asthma Cluster at a Syntactic Foam Manufacturing Facility — Massachusetts 2008–2013

**Published:** 2015-04-24

**Authors:** Megan Casey, Marcia L. Stanton, Kristin J. Cummings, Elise Pechter, Kathleen Fitzsimmons, Ryan F. LeBouf, Christine R. Schuler, Kathleen Kreiss

**Affiliations:** 1 Epidemic Intelligence Service, CDC; 2 Division of Respiratory Disease Studies, National Institute for Occupational Safety and Health, CDC; 3 Occupational Health Surveillance Program, Massachusetts Department of Public Health; 4 Division of Safety Research, National Institute for Occupational Safety and Health, CDC

Work-related asthma is asthma that is caused or exacerbated by exposure to specific substances in the workplace (1). Approximately 10%–16% of adult-onset asthma cases are attributable to occupational factors, and estimates of asthma exacerbated by work range from 13% to 58% (1–3). During 2008–2012, the Massachusetts Department of Public Health received nine reports of work-related asthma among workers at a facility that manufactured syntactic foam used for flotation in the offshore oil and gas industry. These reports and a request from facility employees led to a CDC health hazard evaluation during 2012–2013 in which CDC reviewed records, toured the facility, and administered a questionnaire to current employees. Investigators found that workers’ risk for asthma increased substantially after hire, possibly because of known asthma triggers (i.e., asthmagens) used in production. The company has since initiated efforts to reduce employee exposures to these substances. This cluster of work-related asthma was identified through CDC-funded, state-based surveillance and demonstrates complementary state and federal investigations.

## Case Report

In March 2007, a man aged 53 years with no history of smoking or respiratory disease other than seasonal allergies began employment as an electrician at the syntactic foam manufacturer described in this report. He installed and repaired machines and wiring above machines throughout the facility. These machines processed epoxy resins, curing agents, and other materials, releasing vapors and dust. He occasionally wore a cartridge respirator. In September 2008, he experienced nasal congestion, dyspnea, wheeze, and a nonproductive cough. Despite treatment for allergies and bronchitis, the respiratory symptoms progressed. After 6 weeks, he received a diagnosis of asthmatic bronchitis and began taking an inhaled steroid and a bronchodilator. The symptoms improved but did not resolve. He noted that he felt worse after several hours at work and better when he was away from work.

Over the next 4 months, the man went to the emergency department on several occasions for dyspnea, wheezing, and chest discomfort. In February 2009, suspecting a workplace chemical as the cause of the symptoms, his pulmonologist recommended he take a medical leave of absence for asthma. His symptoms improved. During June–August 2009, he had no exacerbations requiring emergency department visits.

In September 2009, he returned to work with restrictions in place to help prevent exposure to epoxy resins and curing agents. He wore a respirator and avoided the building that used epoxy resins and curing agents. After 3 days, he began experiencing dyspnea and chest tightness. He continued working, and over the next 15 months, he went to the emergency department four times for acute asthma exacerbations. In November 2010, he left his job because of his work-related symptoms. Since leaving, his respiratory symptoms have greatly improved. He still complains of dyspnea when breathing cold air; otherwise, his activities of daily living are not limited. He uses his asthma inhaler 2–3 times per year, representing a large reduction in his inhaler dependence.

## Workplace Investigation

In 2012, a CDC health hazard evaluation was requested by employees of a facility that manufactured syntactic foam used for flotation in the offshore oil and gas industry. In addition, the Massachusetts Department of Public Health recognized a cluster of work-related asthma in their state-based surveillance. During 2008–2012, the department had received nine reports of work-related asthma among workers at the same facility. These cases were reported by six different physicians through the state’s work-related asthma surveillance program, which is supported by CDC. CDC investigators toured the facility to learn about the work processes and conditions and interviewed some production managers, safety managers, and current and former employees. CDC reviewed safety data sheets, injury logs, and medical records and interviewed physicians about illness and exposures among the workers. Known chemical asthmagens were used in the production processes at the facility. In August 2013, all current employees were invited to participate in an interviewer-administered, interpreter-assisted health and work history questionnaire. Using data from the questionnaire, the incidence densities of self-reported adult-onset asthma diagnosed by a physician before and after hire were estimated using birth date, hire date, and diagnosis date. Asthma incidence density before hire was calculated by adding the number of adult-onset asthma diagnoses that occurred before hire and dividing by the sum of participants’ years at risk before hire. Asthma incidence density after hire was calculated by adding the number of adult-onset asthma diagnoses that occurred after hire and dividing by the sum of participants’ time at risk after hire. An incidence ratio was calculated using Poisson regression. Asthma-like symptoms were defined as a response of “yes” to any of the following questions (4):

1. “Are you currently taking any medicine (including inhalers, aerosols or tablets) for asthma?”
2. “Have you had wheezing or whistling in your chest at any time in the last 12 months?”
3. “Have you woken up with a feeling of tightness in your chest at any time in the last 12 months?”
4. “Have you been woken by an attack of shortness of breath at any time in the last 12 months?”

Symptoms that improved when the employees were away from work, either on their days off or when they were on vacation, were considered work related.

A total of 154 (93%) current employees completed the questionnaire. Respondents were primarily men (97%) and foreign born (69%), with a median age of 40 years (range: 21–69 years) and median work tenure at the facility of 5 years (range: <1–21 years). Most worked in production or production support (92%), and the remainder worked in administrative positions. Nine percent (14 of 154) reported receiving a diagnosis of asthma from a physician, and 5% (7 of 154) reported current asthma (Table). Eight of the 14 persons had onset as an adult (i.e., age >18 years), and six of the eight reported receiving a diagnosis of asthma after hire. Available data suggested these six cases had not been previously reported to the Massachusetts Department of Public Health. Adult-onset asthma incidence was 12 times higher (95% confidence interval: 2.3–57.5; p = 0.003) after hire (n = 6; 8.3 cases/1,000 person-years) than before hire (n = 2; 0.7 cases/1000 person-years). Thirty-six (23%) of all respondents reported asthma-like symptoms, the majority (61%) of which had a work-related pattern (Table). Asthma-like symptoms were reported more frequently by those with longer tenure (Figure). Among the 140 respondents without asthma diagnosed by a physician, 27 (19%), or one in five, reported asthma-like symptoms, and 16 (11%) had symptoms that were work related.

### Discussion

This report highlights several important features of work-related asthma, including 1) the temporal relationship between work and symptoms facilitating diagnosis and 2) the frequently ineffective measure of exposure reduction in contrast to the effective measure of complete exposure cessation (6,7). Early identification of affected workers is important because total removal from continued exposure can result in a resolution of asthma symptoms. For example, in one study of workers exposed to an epoxy resin containing methyltetrahydrophthalic anhydride (MTHPA), sensitized workers (i.e., with specific serum immunoglobulin E antibodies to MTHPA) who permanently left their place of employment experienced reduced bronchial reactivity and became symptom-free, whereas workers who stayed experienced no such improvement, despite a tenfold reduction in workplace exposures (8).

The cluster of work-related asthma cases in this report was identified through state-based surveillance funded by CDC. Since 1987, CDC has funded a limited number of state health departments to develop programs for state-based and condition-specific occupational disease and injury surveillance. Diagnosed cases of work-related asthma can act as sentinel events to trigger a public health investigation and intervention (5).

This workplace investigation identified probable additional asthma cases diagnosed by physicians and revealed additional asthma-like symptoms that could represent undiagnosed asthma among coworkers. Although a specific cause was not identified, many potential causes of asthma existed in the facility. Amines and anhydrides found in epoxy resin systems can act as chemical sensitizers by causing allergic reactions (both immediate and delayed) and asthma (8,9). In addition, workers might have been exposed to irritant causes of work-related asthma. Thus, various substances could have contributed to respiratory symptoms in this facility.

In response to the findings of the investigation, CDC recommended enhanced engineering controls, completing the proposed respiratory protection program, and improved communication about hazards through use of signs in native languages of the employees. Based on these recommendations, the company upgraded equipment in the facility, installed a dust collection system, and reduced manual handling of chemicals in the tumbling machine area. A mandatory respiratory protection program in this area also was implemented.

The findings in this report are subject to at least two limitations. First, the health and work history questionnaire was administered to current workers only. These workers might have been healthier than all workers who had ever been employed at the facility because workers who were too ill to work might have resigned, possibly resulting in an underestimation of work-related asthma. Second, CDC investigators relied on self-reported health concerns and whether symptoms were work related, responses that might be subject to recall or reporting bias.

Occupational risk factors should be considered during assessments of patients with asthma-like symptoms and those with existing asthma. Only one in seven employed adults with asthma talk to their clinician about the possible role of work in their disease (10). Physician recognition of work-related respiratory symptoms might allow workers to recover by eliminating exposure to the substances causing the illness. Physician reporting of work-related illnesses is vital to the success of occupational surveillance.

What is already known about this topic?

Work-related asthma is common but is underrecognized and underreported by clinicians. Early diagnosis of work-related asthma and subsequent cessation of exposure to substances that cause asthma can lead to resolution of asthma symptoms among workers with existing asthma and can prevent future cases.

What is added by this report?

This cluster of work-related asthma was identified by CDC-funded, state-based surveillance. A CDC investigation identified additional asthma cases diagnosed by physicians and revealed additional asthma-like symptoms that could represent undiagnosed asthma among coworkers. Adult-onset asthma incidence was 12 times higher after hire than before hire.

What are the implications for public health practice?

Diagnosed cases of work-related asthma can be sentinel events that trigger public health investigations and interventions. Occupational risk factors should be considered among patients with asthma-like symptoms and those with existing asthma. Sentinel occupational health surveillance can be an important tool for identifying emerging work-related risks.

## Figures and Tables

**FIGURE:**
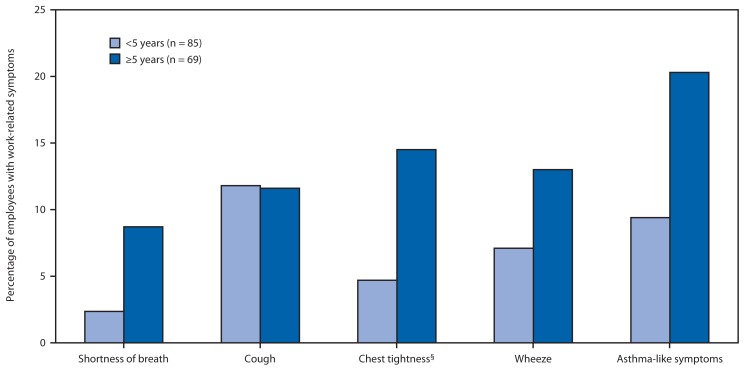
Prevalence of work-related respiratory symptoms* among employees^†^ of a syntactic foam manufacturing facility, by number of years at the facility — Massachusetts, August 2013 * Asthma-like symptoms were defined as a response of “yes” to any of the following questions: “Are you currently taking any medicine (including inhalers, aerosols or tablets) for asthma?”“Have you had wheezing or whistling in your chest at any time in the last 12 months?”“Have you woken up with a feeling of tightness in your chest at any time in the last 12 months?”“Have you been woken by an attack of shortness of breath at any time in the last 12 months?” Work-related symptoms improved when the employees were away from their workplace, either when the employees had days off or were on vacation. ^†^ N = 154. ^§^ Statistically significant difference between tenure groups (p<0.05).

**TABLE:** Self-reported respiratory symptoms and asthma diagnoses among current workers* at a syntactic foam manufacturer — Massachusetts, August 2013

Symptom or asthma diagnosis	Overall		Work related†	
	
	No.	(%)	No.	(%)
**Symptom (in last 12 months)**				
Shortness of breath	**13**	**(8)**	8	(5)
Cough	**38**	**(25)**	18	(12)
Wheeze	**23**	**(15)**	15	(10)
Chest tightness	**20**	**(13)**	14	(9)
Burning throat	**21**	**(14)**	17	(11)
Asthma attack	**5**	**(3)**	2	(1)
Asthma-like symptoms§	**36**	**(23)**	22	(14)
**Asthma diagnosis (ever)** ¶	**14**	**(9)**	—	—
Adult onset**	**8**	**(5)**	—	—
After hire**	**6**	**(4)**	—	—

* N = 154.

† Work-related symptoms were defined as symptoms that improved when the employees were away from their workplace, either when the employees had days off or were on vacation.

§ Asthma-like symptoms were defined as a response of “yes” to any of the following questions (**Source:** Grassi M, Rezzani C, Biino G, Marinoni A. Asthma-like symptoms assessment through ECRHS screening questionnaire scoring. J Clin Epidemiol 2003;56:238–47):
“Are you currently taking any medicine (including inhalers, aerosols or tablets) for asthma?” “Have you had wheezing or whistling in your chest at any time in the last 12 months?” “Have you woken up with a feeling of tightness in your chest at any time in the last 12 months?” “Have you been woken by an attack of shortness of breath at any time in the last 12 months?”

¶ Respondents who ever received a diagnosis of asthma responded “yes” to the question: “Has a physician ever told you that you have asthma?” Adult-onset asthma cases were diagnosed among persons aged >18 years.

** Categories of adult-onset and after hire are not mutually exclusive. Some respondents might be reflected in both categories.
